# Low EBI3 Expression Promotes the Malignant Degree of Gastric Cancer

**DOI:** 10.1155/2022/5588043

**Published:** 2022-03-17

**Authors:** Qiqi Gu, Han Wu, Zhouyang Cheng, Xiaofei Zhi, Qingjie Song, Xiaoyang Chen, Xiaobing Yang

**Affiliations:** ^1^Department of General Surgery, The Affiliated Hospital of Nantong University, Jiangsu Province 226001, China; ^2^Medical College of Nantong University, Nantong, Jiangsu Province 226001, China; ^3^Department of Ultrasound, The Affiliated Hospital of Nantong University, Jiangsu Province 226001, China; ^4^Department of General Surgery, Huaian Hospital of Huaian City, Jiangsu Province 223200, China

## Abstract

**Objective:**

To explore expression changes and clinical significance of EBI3 in gastric cancer.

**Methods:**

Expression of EBI3 in gastric cancer (GC) cell lines, GC tissues, and corresponding adjacent tissues were detected by qRT-PCR, Western blot, or immunohistochemistry. The relationship between the EBI3 expression and clinicopathological features of GC patients was analyzed. Expression of EBI3 in BGC-823 was overexpressed or downregulated, then, the changes of proliferation, migration, invasion, and tumorigenicity of BGC-823 were observed by MTT, scratch test, Transwell test, and tumorigenesis assay model.

**Results:**

EBI3 was lowly expressed in GC tissues. EBI3 expression in BGC823 was highest than other cell lines. EBI3 expression was significantly associated with TNM stage. GC patients with low expression of EBI3 had a rather poor prognosis than the GC patients with high expression of EBI3. Low EBI3 expression was an independent risk predictor of the prognosis of GC patients. After EBI3 was overexpressed, the viability, migration, invasion, and tumorigenicity abilities of BGC-823 were significantly reduced. Opposite effect was observed after EBI3 expression was downregulated.

**Conclusion:**

EBI3 low expression is closely related to the malignant degree of GC and may be a predictive indicator of the prognosis of GC and potential therapeutic targets.

## 1. Introduction

Gastric cancer (GC) is one of the most common malignant tumors in the world. Its morbidity rate ranks fourth among malignant tumors and its case fatality rate ranks second. The number of newly discovered GC patients in the world is about 1 million each year. About 700,000 people die of GC every year [1–4]. The early diagnosis rate of GC is low, and most patients have entered the advanced stage after diagnosis. Metastasis and recurrence of gastric cancer are the main causes of its death [5–7]. With the rapid development of molecular biology, many studies are devoted to finding key molecules in the process of tumor invasion and metastasis and controlling tumor metastasis by interfering with these molecules. It is of great significance to find new therapeutic targets for the effective treatment of GC patients and to improve the survival rate and clinical prognosis of GC patients.

Epstein-Barr virus-induced Gene 3 (EBI3), as a soluble cytokine receptor, is a homolog of the p40 subunit of the IL-12 family. It was originally discovered in products secreted by EB virus-infected B lymphocytes [8]. In recent years, studies have found that EBI3 has abnormal expression in a variety of malignant tumors, and the role of EBI3 in different malignant tumors is also different. It was found that abnormal expression of EBI3 was observed in Hodgkin lymphoma [9], nasopharyngeal carcinoma [10], malignant melanoma [11], lung cancer [11], colon cancer [12], pancreatic cancer [13], gastric cancer [14], acute myeloid leukemia [15], and various tumor cell lines [16], and EBI3 also played an important regulatory role in the formation and development of tumors.

Therefore, what kind of role EBI3 plays in malignant tumors, whether it can become a new effective therapeutic target for tumors, and whether it can become a breakthrough in overcoming tumors, has gradually attracted people's attention. In this research, the expression of EBI3 in GC cell lines, GC tissues, and the corresponding adjacent tissues was detected. The relationship between the EBI3 expression and clinicopathological features of GC patients was analyzed. The expression of EBI3 in BGC-823 was overexpressed or downregulated, then, the changes of the proliferation, migration, and invasion abilities of BGC-823 were observed. This research is aimed at exploring the expression changes and clinical significance of EBI3 in GC.

## 2. Material and Methods

### 2.1. Patients and Tissue Samples

In this experiment, a total of 353 GC patients who underwent GC D2 radical surgery were randomly selected in the Department of General Surgery of the Affiliated Hospital of Nantong University from March 2004 to March 2011. 353 pair's tissue microarray specimens were obtained. Thirty pairs of fresh tissue specimens were from GC patients who underwent D2 radical surgery in the Department of General Surgery of the Affiliated Hospital of Nantong University from October 2015 to March 2016. The corresponding tissues adjacent to cancer beyond 5.0 cm were taken as the control. All patients were diagnosed as GC by pathological examination for the first time and did not receive any anticancer treatment before surgery. This study was approved by the Ethics Committee of the Affiliated Hospital of Nantong University, and the patients signed a written informed consent. The final follow-up for all cases in this study was March 31, 2016. The clinical baseline data of the cases were shown in Table 1.

### 2.2. Quantitative Real-Time RT-PCR (qPCR)

The total RNA of tissues or cells was isolated using UNIQ-10 Spin Column RNA Purified Kit (Sangon, China). The RevertAidTM First Strand cDNA Synthesized Kit (Fermentas, Canada) was used to synthesize the first-strand cDNA, and the synthesized cDNA was subsequently subjected to Corbett RG-6000 PCR system (QIAGEN, German) using FastStart Universal SYBR Green Master Mix (Roche, Switzerland). The sense and antisense primers were synthesized as follows: GAPDH 5′-ACTCTTCCAGCCTTCCTTCCT-3′, 5′-CAGTGATCTCCTTCTGCATCCT-3′; EBI3 5′-TTACAAGCGTCAGGGAGCTG-3′, 5′-TTCCCCGTAGTCTGTGAGGT-3′. The EBI3 mRNA level was analyzed with 2^-*Δ*CT^ method.

### 2.3. Western Blot

The total protein was extracted using RIPA buffer with 100 mM of phenylmethylsulphonyl fluoride as a protease inhibitor (Beyotime, China). Briefly, the protein was transferred to the PVDF membrane, and then, the PVDF membrane was successively incubated with the primary antibodies and second antibodies as follows: 1 : 400 diluted rabbit anti-EBI3 antibody (SIGMA, USA), 1 : 1500 diluted mouse anti-*β*-actin antibody (KANGCHEN, China), 1 : 5,000 IRDye diluted 700-conjugated affinity-purified goat anti-mouse antibody (Rockland Immunochemicals, USA), or 1 : 5,000 diluted IRDye 800-conjugated affinity-purified goat anti-rabbit antibody (Rockland Immunochemicals, USA). The protein bands were visualized using Odyssey laser scanning system (LI-COR Inc., USA), and the intensities were quantified by Odyssey 3.0 image analysis system software.

### 2.4. Immunohistochemistry (IHC)

The matched cancer tissues and adjacent normal tissues of 353 cases of GC patients were selected for paraffin embedding and tissue microarray. Briefly, the slides were incubated with primary and second antibodies successively as follows: rabbit Anti-EBI3 (1 : 2500, SIGMA, USA) and HRP-labeled goat anti-rabbit IgG (1 : 1500, Santa Cruz, USA). Then, the DAB was used for staining.

Double-blind readings were performed by two senior pathologists. Staining intensity was analyzed as followed: “0”: refers to those without staining in the cytoplasm; “1”: refers to the cytoplasm staining was light yellow; “2”: refers to the cytoplasm staining was brownish yellow; “3 points”: refers to the cytoplasm staining was deep yellow. Then, the EBI3 positive rate on each tissue chip was calculated. Take the product of the score of staining intensity and the EBI3 positive rate as the score of EBI3 expression intensity. We took the median value of 150 as the cutoff value, and according to the expression level of EBI3, we divided it into a low expression group (0 to 150 points) and a high expression group (151 to 300 points).

### 2.5. GC Cell Line Culture

Gastric cancer cell lines MKN-45, MKN-28, MGC-803, HGC-27, and BGC-823 from biological sample bank of the Affiliated Hospital of Nantong University were cultured in RPMI-1640 medium (contains 10% fetal bovine serum) (Gibco BRL, USA) in 37°C, 5% CO₂ incubator.

### 2.6. EBI3 of BGC-823 Cells Were Overexpressed or Downregulated

EBI3 siRNA plasmid or overexpression lentivirus was constructed by Guangzhou Lvbo Biotechnology Co., Ltd. BGC-823 cells were cultured in a 6-well culture plate at a density of 4 × 10^4^/ml in a 37°C, 5% CO_2_ incubator. When the cells grow to a density of about 70% to 80%, transfection experiments were performed with LipofectamineTM2000 (Invitrogen, USA). The EBI3 expression level in BGC-823 cells was confirmed by RT-PCR and Western blot.

### 2.7. MTT Assay

The above transfected BGC-823 cells were cultured in the 96-well culture plate at a density of 4 × 10^4^/ml in a 37°C, 5% CO_2_ incubator overnight. When the cells grew to a density of about 90%, carefully aspirated the medium, added 100 *μ*l of 5 mg/ml MTT mixture to each well, incubated at 37°C for 4 h, removed the supernatant, added 100 *μ*l DMSO to each well, and then used multifunctional microplate reader to detect OD value at 0 h, 24 h, 48 h, and 72 h.

### 2.8. Cell Scratch Test

The above-transfected cells were cultured in the 6 cm culture plate at a density of 4 × 10^4^/ml in a 37°C, 5% CO_2_ incubator, and when the cells grew to substantially cover the bottom of the plate, the cells were scratched vertically with a sterile 100 *μ*l pipette tip. Washed off the detached cells with sterile PBS, and then continued to culture for 48 hours and detect the repair of scratches at 0, 24, and 48 hours, respectively. Scratch repair rate = (initial scratch width − width during inspection)/initial scratch width.

### 2.9. Transwell Assay

The above-transfected cells were seeded at a density of 4 × 10^4^ in the upper chamber coated with a layer of Matrigel (50 *μ*l/cm^2^) and cultured with serum-free RPMI-1640 medium, and then the lower chamber was added with RPMI-1640 medium containing 10% fetal bovine serum. The cells were cultured for 48 hours, and then the migrated cells on the lower surface of the upper chamber were fixed with 4% paraformaldehyde for 15 minutes and stained with purple crystals, then observed under a fluorescence microscope (Leica, Germany).

### 2.10. Tumorigenesis Assay Model

All animal model experiment was performed according to protocols approved by the Institutional Animal Care and Use Committee at Nantong University. The 0.1 ml (density of 5 × 10^6^) transfected cells were injected subcutaneously in the back of the neck of Athymic male nude 4- to 5-week-old BALB/c-nu/nu mice. Tumors' weight was measured after 4 weeks.

### 2.11. Statistical Analysis

The data of this research was processed and analyzed by SPSS19.0 statistical software. The measurement data were expressed as mean ± SD, and the Cox proportional hazards model was used for multivariate analysis of variables that are meaningful for single-factor analysis. The survival rate curve was analyzed by the Kaplan-Meier method, and comparison between groups was carried out by the log-rank method. The relationship between the expression of EBI3 and the clinicopathological characteristics was analyzed by chi-square test or Fisher exact probability method. One-way analysis of variance (ANOVA) was used for comparison between groups. The difference was statistically significant when *P* < 0.05.

## 3. Results

### 3.1. EBI3 Expression in GC Cell Lines and GC Tissues

**Figure 1:**
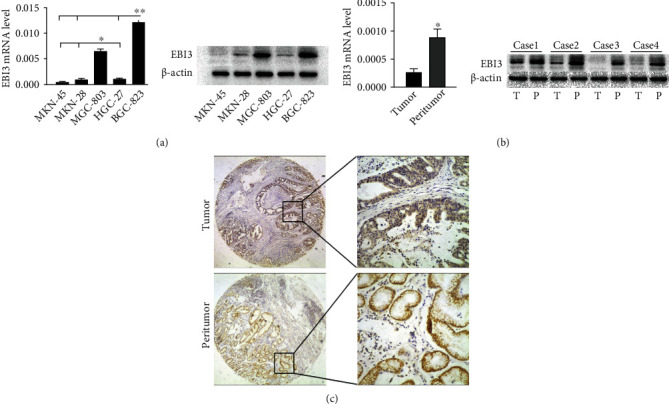
EBI3 expression in gastric cancer (GC) cell lines and GC tissues. (a) The mRNA expression of EBI3 in GC cell lines MKN-45, MKN-28, MGC-803, HGC-27, and BGC-823 was detected, and the expression level of EBI3 was different in each GC cell line. The expression level was the lowest in MKN-45 and was significantly higher in BGC-823 and MGC-803 than other GC cell lines. (b) The mRNA and protein expression levels of EBI3 in gastric cancer tissues were significantly lower than those in paratumor tissues. (c) EBI3 expression was detected in tissue microarray by IHC. The expression levels of EBI3 in GC tissues were lower than that in paratumor tissues. The left column, ×40; the right column, ×200. ^∗^*P* < 0.05, ^∗∗^*P* < 0.01.

The expression of in GC cell lines MKN-45, MKN-28, MGC-803, HGC-27, and BGC-823 was detected, and we found that the expression level of EBI3 mRNA was different in each GC cell line. The EBI3 expression level was the lowest in MKN-45 and was significantly higher in BGC-823 and MGC-803 than other gastric cancer cell lines (*P* < 0.05) (Figure 1(a)). We selected BGC-823 as the follow-up research object. To detect the expression level of EBI3 in GC tissues, we used RT-PCR and Western blot to detect the expression levels of EBI3 mRNA and protein in 30 matched cancer tissues and adjacent tissues. It was found that the expression levels of EBI3 mRNA and protein in cancer tissues were significantly lower than those in adjacent tissues (*P* < 0.05) (Figure 1(b)). In addition, we detected the expression of EBI3 in 353 cases of GC paired tissue microarray by IHC method. The EBI3 protein was mainly expressed in the cytoplasm. We found that EBI3 was in low expression in GC tissues and high expression in corresponding paracancerous tissues (Figure 1(c)).

### 3.2. Relationship between EBI3 Expression and Clinicopathological Characteristics of GC

According to the results of tissue microarray detection, 191 of 353 GC patients with EBI3 low expression and 162 with EBI3 high expression, and the relationship between EBI3 expression and clinicopathological characteristics of GC was analyzed by chi-square test or Fisher exact probability method. The results showed EBI3 expression was significantly associated with TNM stage, T, N, M (*P* < 0.05), and not related to the patient's gender, age, histological type, differentiation, CEA, CA199, etc. (*P* > 0.05) (Table 1).

### 3.3. Relationship between the Expression of EBI3 and the Prognosis of Patients with GC

The results of univariate analysis using the Kaplan-Meier method showed that the overall survival (OS) of patients was closely related to differentiation, TNM stage, T, N, M, and EBI3 (*P* < 0.05) (Table 2). Multivariate survival analysis using the Cox proportional hazards model showed that EBI3, differentiation, T, N, and M are independent prognostic factors for patients with gastric cancer (*P* < 0.05) (Table 3).

**Figure 2:**
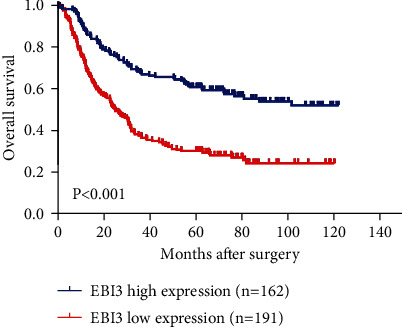
The prognostic value of EBI3 in 353 GC patients after surgery. The overall survival of GC patients with low expression of EBI3 was significantly decreased after the operation, compared with those with high EBI3 expression.

Kaplan-Meier method was used to analyze the relationship between the expression level of EBI3 and the survival status of 353 GC patients after surgery. Through the OS curve analysis, we found that the OS of patients with low expression of EBI3 group was significantly decreased after the operation, compared with the high expression of the EBI3 group (*P* < 0.05) (Figure 2).

### 3.4. EBI3 of BGC-823 Cells Were Overexpressed or Downregulated

**Figure 3:**
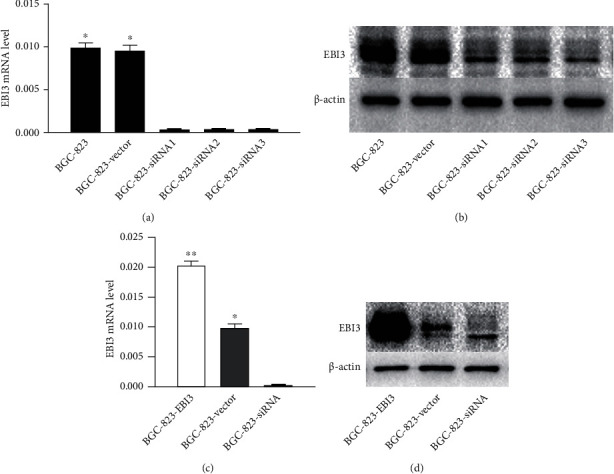
EBI3 of BGC-823 cells was overexpressed or downregulated. (a, b) After siRNA interferes with BGC-823, the total RNA and protein were extracted and detected by RT-PCR and Western blot. The interference effect of siRNA3 was the best and be selected as the interference fragment for subsequent experiments. (c) The overexpression group (BGC-823-EBI3) EBI3 mRNA expression level was significantly increased, and the interference group (BGC-823-EBI3-siRNA) EBI3 mRNA expression level was significantly decreased. (d) The EBI3 protein detection with Western blot was consistent with RT-PCR. ^∗^*P* < 0.05. ^∗∗^*P* < 0.01.

After using LipofectamineTM2000-siRNA fragment to interfere with BGC-823 for 24 hours, the total RNA and protein were extracted and detected by RT-PCR and Western blot. The results showed that the interference effect of siRNA3 was the best (*P* < 0.05), then selected siRNA3 as the interference fragment for subsequent experiments (Figures 3(a) and 3(b)). Through RT-PCR, it was found that compared with the control group (BGC-823-vector), the overexpression group (BGC-823-EBI3) EBI3 mRNA expression level was significantly increased, the interference group (BGC-823-EBI3-siRNA) EBI3 mRNA expression level was significantly decreased, and the difference was statistical significance (*P* < 0.05). The results of EBI3 protein detection on Western blot were consistent with RT-PCR (Figures 3(c) and 3(d)).

### 3.5. EBI3 Low Expression Promoted the Viability, Migration, Invasion, and Tumorigenicity Abilities of BGC-823 Cells

**Figure 4:**
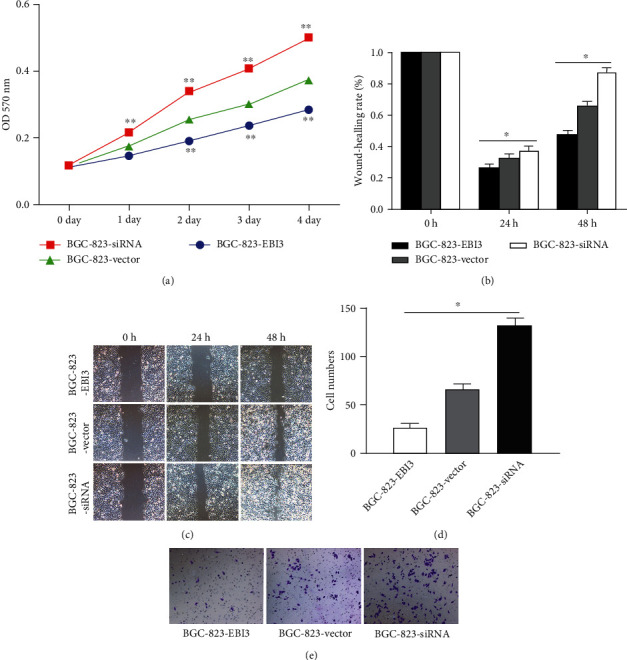
EBI3 low expression promoted the viability, migration, invasion, and tumorigenicity abilities of BGC-823 cells. (a) The MTT assay showed that compared with the control group, the cell viability of the EBI3 overexpression group was significantly reduced, and the cell viability of the EBI3 interference group increased significantly. (b, c) After the cell scratched, the repair rate of the interference group was significantly faster than that of the control group, and the repair rate of the overexpression group was significantly slower than that of the control group. (d, e) The invasion experiments showed that compared with the control group, the invasive ability of BGC-823 cells in the overexpression group was inhibited, and the number of cell penetration was the least, while the invasive ability of BGC-823 cells in the interference group was significantly enhanced, and the number of cell penetration was the largest. ^∗^*P* < 0.05. ^∗∗^*P* < 0.01.

The results of the MTT assay showed that, compared with the control group, the cell viability of the EBI3 overexpression group was significantly reduced (*P* < 0.05), indicating that EBI3 overexpression inhibited the viability of BGC-823 cells. The cell viability of the EBI3 interference group increased significantly (*P* < 0.05). It showed that the low expression of EBI3 promoted the activity of BGC-823 cells (Figure 4(a)).

After the cell scratched, the repair rate of the interference group was significantly faster than that of the control group, and the repair rate of the overexpression group was significantly slower than that of the control group (*P* < 0.05), indicating that interference with EBI3 expression promoted the migration ability of BGC-823 cells, and overexpression of EBI3 inhibited BGC-823 cell migration ability (Figures 4(b) and 4(c)).

The results of cell invasion experiments showed that, compared with the control group, the invasive ability of BGC-823 cells in the overexpression group was inhibited, and the number of cell penetration was the least (*P* < 0.05), while the invasive ability of BGC-823 cells in the interference group was significantly enhanced, and the number of cell penetration was the largest (*P* < 0.05) (Figures 4(d) and 4(e)).

**Figure 5:**
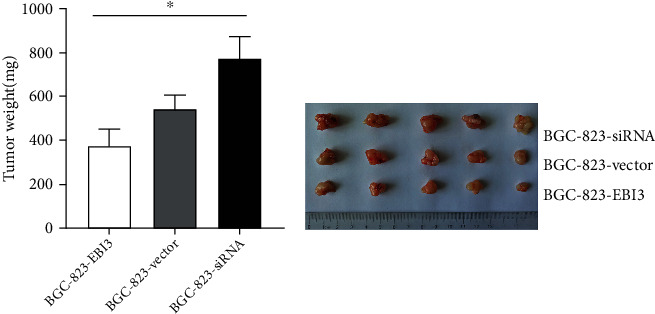
The tumorigenesis assay experiments. Compared with the control group, the tumors' weight reduced in the overexpression group and increased in the interference group, and the difference was statistically significant. ^∗^*P* < 0.05.

The results of tumorigenesis assay experiments showed that, compared with the control group, the tumors' weight reduced in the overexpression group and increased in the interference group, and the difference was statistically significant (*P* < 0.05) (Figure 5). The results indicated that interference with EBI3 expression promoted the tumorigenicity ability of BGC-823 cells, and overexpression of EBI3 inhibited BGC-823 cell tumorigenicity ability.

## 4. Discussion

In China, the incidence of GC accounts for the second-highest rate of gastrointestinal malignant tumors, and the death rate of GC patients accounts for the second highest among patients with malignant tumor deaths [3]. With the continuous advancement of medical technology, advanced medical examination instruments and equipment are used in clinics, the level of diagnosis and treatment of GC has been improved to a certain extent, and the 5-year survival rate of GC patients has increased [17, 18]. However, due to the lack of specific clinical symptoms of early GC patients and the lack of convenient screening methods, the diagnosis rate of early GC is relatively low, and most of the GC patients have entered the middle and late stages after diagnosis [19]. The prognosis of most GC patients is still unsatisfactory. GC patients have a higher rate of recurrence and metastasis after surgery, which seriously affects the treatment effect of GC [20, 21]. Therefore, if a predictive factor that predicts the prognosis of GC patients can be found and used as an effective treatment target, it has important significance for improving the clinical prognosis of GC patients.

In this research, the expression level of EBI3 in GC tissues was detected by RT-PCR, Western blot, and IHC, and the results showed EBI3 was lowly expressed in GC tissues. Then, the relation between EBI3 expression and GC was analyzed, and the results showed EBI3 expression significantly associated with the TNM stage. GC patients with low expression of EBI3 had a rather poor prognosis than the GC patients with high expression of EBI3, and low EBI3 expression was an independent risk predictor of the prognosis of GC patients. It has been reported that the abnormal expression of EBI3 is closely related to the poor prognosis of many malignant tumors, such as lung cancer [11], colon cancer [12], and nasopharyngeal cancer [10], and EBI3 plays different roles in different tumors. In a colon cancer study, Zeng et al. [22] observed that patients with high EBI3 expression had poor clinical prognosis. In a study of lung cancer, Nishino et al. [23] found that the higher the EBI3 expression level, the worse the clinical prognosis of patients. The above two research results are completely opposite to our observations. To further observe the effect of changes in the expression of EBI3 on the biological characteristics of GC cells, we selected BGC-823 cells as observation objects. After EBI3 overexpression or downregulation, the cell viability, migration, invasion, and tumorigenicity ability were observed, and the results showed that EBI3 low expression promoted the viability, migration, invasion, and tumorigenicity ability of BGC-823 cells, and overexpression of EBI3 inhibited BGC-823 cell viability, migration, invasion, and tumorigenicity ability. This result is consistent with Long et al.'s study [16] on the effect of EBI3 on human liver cancer cells. Why EBI3 exerts completely opposite effects on different tumors is still unclear. Some scholars believe that EBI3 can regulate the differentiation of immune cells and the expression of related cytokines. Through the immunoregulatory mechanisms, it participates in different tumor development processes and thus exhibits different biological effects [24]. The specific mechanism still needs further research and clarification.

Although IL-35 was found playing an antitumorigenic role in several types of cancer, accumulating evidence suggests that increased levels of IL-35 can be correlated with the pathogenicity and progression of cancer. Mirlekar and Pylayeva-Gupta [25] found that IL35 is an important immunosuppressive driver in pancreatic ductal adenocarcinoma (PDA) and potentiates tumor growth via the suppression of endogenous antitumor CD4^+^ effector T cell responses. They also demonstrated that B cell-specific deletion of IL35 could facilitate CD8^+^ T cell activation in PDA. It is reported that the expression of IL-12p35, one of subunits of IL-35, is not been detected in the HCC tissues. In addition, although IL-12p35 positive expression was associated with a worse survival in nasopharyngeal carcinoma, multivariate analyses suggested EBI3 rather than IL-12p35 was an independent prognostic marker [26]. So we assumed that EBI3 may be as a main functional subunit and plays a major role in IL-35 and has different impacts on prognosis based on tumor type.

In spite of our findings, limitations of our research need to be addressed. First, it is a retrospective observational study. Thus, the findings might not be applicable to the general population. Larger prospective studies are needed to confirm our results. Second, the IHC data are semiquantitative; additional methods are needed to evaluate and confirm the rates of EBI3 expression in tumor cells. In addition, further studies are necessary to investigate the underlying mechanisms by which EBI3 influences the invasion and metastasis of gastric cancer cells.

In summary, our results indicated that EBI3 low expression is closely related to the malignant degree of GC and may be a predictive indicator of the prognosis of GC and a potential therapeutic target.

## Figures and Tables

**Table 1:** Relationship between EBI3 expression and clinicopathological characteristics of GC patients.

Characteristic	*n* (*n* = 353)	EBI3-(*n* = 191)	EBI3+(*n* = 162)	*χ* ^2^	*P* value
Gender				0.228	0.633
Male	266	142 (53.4%)	124 (46.6%)		
Female	87	49 (56.3%)	38 (43.7%)		
Age				0.27	0.869
<60	128	70 (54.7%)	58 (45.3%)		
≥60	225	121 (53.8%)	104 (46.2%)		
Histological type				3.666	0.300
Tubular	8	3 (37.5%)	5 (62.5%)		
Mixed and mucinous	94	45 (47.9%)	49 (54.1%)		
Signed ring cell	185	103 (55.7%)	82 (44.3%)		
Others	66	40 (60.6%)	26 (39.4%)		
Differentiation				2.950	0.399
Well	10	3 (30.0%)	7 (70.0%)		
Middle	141	74 (52.5%)	67 (47.5%)		
Poor	182	103 (56.6%)	79 (43.4%)		
Others	20	11 (55.0%)	9 (45.0%)		
TNM stage				29.840	<0.001^∗^
0	7	0 (0.0%)	7 (100.0%)		
Ia + Ib	54	19 (35.2%)	35 (64.8%)		
IIa + IIb	127	63 (49.6%)	64 (50.4%)		
IIIa + IIIb	134	84 (62.7%)	50 (47.3%)		
IIIc + IV	31	25 (80.6%)	6 (19.4%)		
T				19.951	0.001^∗^
Tis	7	0 (0.0%)	7 (100.0%)		
T1	26	9 (34.6%)	17 (65.4%)		
T2	72	32 (44.4%)	40 (55.6%)		
T3	219	130 (59.4%)	89 (40.6%)		
T4	29	20 (69.0%)	9 (31.0%)		
N				19.039	<0.001^∗^
N0	120	49 (40.8%)	71 (59.2%)		
N1	67	36 (53.7%)	31 (46.3%)		
N2	76	42 (55.3%)	34 (44.7%)		
N3	90	64 (71.1%)	26 (28.9%)		
M				3.276	0.070
M0	331	175 (52.9%)	156 (47.1%)		
M1	22	16 (72.7%)	6 (27.3%)		
Preoperative CEA (ng/ml)				1.132	0.568
≤5	119	63 (52.9%)	56 (47.1%)		
>5	42	20 (47.6%)	22 (52.4%)		
Unknown	192	108 (56.3%)	84 (43.7%)		
Preoperative CA199 (U/ml)				0.919	0.632
≤37	126	64 (50.8%)	62 (49.2%)		
>37	26	14 (53.8%)	12 (46.4%)		
Unknown	201	113 (56.2%)	88 (43.8%)		

Statistical analyses were performed by the Pearson *χ*^2^ test. TNM stage: tumor node metastasis stage, CEA: carcinoembryonic antigen, CA199: carbohydrate antigen 19-9. ^∗^*P* < 0.05 is considered significant.

**Table 2:** Univariate analysis of the overall survival rate of GC patients.

Variables	Univariate		
	HR	*P* > ∣*z*∣	95% CI
Gender			
Male vs. female	0.888	0.452	0.651-1.210
Age (years)			
≤60 vs. >60	1.184	0.248	0.889-1.576
Histological type			
Tubular vs. mixed (tubular and mucious) and mucinous vs. signet cell carcinoma	0.950	0.402	0.842-1.071
Differentiation			
Well vs. middle vs. poor	1.497	<0.001^∗^	1.201-1.867
TNM stage			
0 vs. Ia + Ib vs. IIa + IIb vs. IIIa + IIIb vs. IIIc + IV	2.271	<0.001^∗^	1.914-2.695
T			
Tis vs. T1 vs. T2 vs. T3 vs. T4	1.818	<0.001^∗^	1.486-2.224
N			
N0 vs. N1 vs. N2 vs. N3	1.635	<0.001^∗^	1.451-1.843
M			
M0 vs. M1	4.230	<0.001^∗^	2.647-6.762
Preoperative CEA, ng/ml			
≤5 vs. >5	1.034	0.499	0.938-1.139
Preoperative CA199, U/ml			
≤37 vs. >37	1.007	0.891	0.916-1.107
EBi3 expression			
High vs. low	0.402	<0.001^∗^	0.315-0.561

The univariate analysis was performed by Kaplan-Meier method with log-rank test. ^∗^*P* < 0.05 is considered significant.

**Table 3:** Multivariate analysis of the overall survival rate of GC patients.

Variables	Multivariate		
	HR	*P* > ∣*z*∣	95% CI
EBi3 expression			
High vs. low	0.573	<0.001^∗^	0.426-0.772
Differentiation			
Well vs. middle vs. poor	1.270	0.030^∗^	1.024-1.574
TNM stage			
0 vs. Ia + Ib vs. IIa + IIb vs. IIIa + IIIb vs. IIIc + IV	1.019	0.939	0.627-1.656
T			
Tis vs. T1 vs. T2 vs. T3 vs. T4	1.445	0.027^∗^	1.043-2.002
N			
N0 vs. N1 vs. N2 vs. N3	1.399	0.006^∗^	1.100-1.779
M			
M0 vs. M1	3.091	0.002^∗^	1.492-6.405

Multivariate analysis was performed by the Cox proportional hazards model. ^∗^*P* < 0.05 is considered significant.

## Data Availability

The datasets used or analyzed during the current study are available from the corresponding author on reasonable request.
